# Evaluation of Arsenic Trioxide Potential for Lung Cancer Treatment: Assessment of Apoptotic Mechanisms and Oxidative Damage

**DOI:** 10.4172/1948-5956.1000379

**Published:** 2015-12-18

**Authors:** Alice M Walker, Jacqueline J Stevens, Kenneth Ndebele, Paul B Tchounwou

**Affiliations:** 1 Molecular and Cellular Biology Research Laboratory, Jackson State University, Jackson, Mississippi, USA; 2 Laboratory of Cancer Biology and Target Validation, Jackson State University, Jackson, Mississippi, USA; 3 Molecular Toxicology Research Laboratory, NIH-Center for Environmental Health, College of Science, Engineering and Technology, Jackson State University, Jackson, Mississippi, USA

**Keywords:** Arsenic trioxide, A549 cells, Oxidative stress, Hsp70, c-fos, p53, bcl-2, Apoptosis, Genotoxicity

## Abstract

**Background:**

Lung cancer is one of the most lethal and common cancers in the world, causing up to 3 million deaths annually. The chemotherapeutic drugs that have been used in treating lung cancer include cisplatin-pemetrexed, cisplastin-gencitabinoe, carboplatin-paclitaxel and crizotinib. Arsenic trioxide (ATO) has been used in the treatment of acute promyelocytic leukemia. However, its effects on lung cancer are not known. We hypothesize that ATO may also have a bioactivity against lung cancer, and its mechanisms of action may involve apoptosis, DNA damage and changes in stress-related proteins in lung cancer cells.

**Methods:**

To test the above stated hypothesis, lung carcinoma (A549) cells were used as the test model. The effects of ATO were examined by performing 6-diamidine-2 phenylindole (DAPI) nuclear staining for morphological characterization of apoptosis, flow cytometry analysis for early apoptosis, and western blot analysis for stress-related proteins (Hsp70 and cfos) and apoptotic protein expressions. Also, the single cell gel electrophoresis (Comet) assay was used to evaluate the genotoxic effect.

**Results:**

ATO-induced apoptosis was evidenced by chromatin condensation and formation of apoptotic bodies as revealed by DAPI nuclear staining. Cell shrinkage and membrane blebbing were observed at 4 and 6 µg/ml of ATO. Data from the western blot analysis revealed a significant dose-dependent increase (p < 0.05) in the Hsp 70, caspase 3 and p53 protein expression, and a significant (p < 0.05) decrease in the cfos, and bcl-2 protein expression at 4 and 6 µg/ml of ATO. There was a slight decrease in cytochrome c protein expression at 4 and 6 µg/ ml of ATO. Comet assay data revealed significant dose-dependent increases in the percentages of DNA damage, Comet tail lengths, and Comet tail moment.

**Conclusion:**

Taken together our results indicate that ATO is cytotoxic to lung cancer cells and its bioactivity is associated with oxidative damage, changes in cellular morphology, and apoptosis.

## Background

Lung cancer is one of the most lethal and common of cancers in the world, causing up to 3 million deaths, annually [1,2]. Only one in ten patients diagnosed with lung cancer has a survival of 5 years [3]. It is a leading cause of cancer death in men and women in the United States and more people die from lung cancer than any other type of cancer. The chemotherapeutic drugs that are currently being used in treating lung cancer are cisplatin-pemetrexed, cisplastin-gencitabinoe, carboplatin-paclitaxel and crizotinib [4]. However, the prognosis is still poor despite advances in present therapies. There is still a need for more effective treatment strategies.

Arsenic trioxide (ATO) has been used as an anticancer agent in traditional Chinese medicine for many years. In vitro studies have also demonstrated that ATO exerts its therapeutic mechanisms through a multitude of biochemical events including cell cycle modulation and apoptosis in leukemia cell. Recently, the Food and Drug Administration has approved ATO, the trade name Trisenox as a chemotherapeutic agent for the treatment of relapsed/refractory acute promyelocytic leukemias, head and neck cancer neuroblastoma [5–8].

Apoptosis is an active and gene–directed form of cell death. The role of apoptosis is to maintain tissue homeostasis and to eliminate excess or dysfunctional cells. Its biochemical features include activation of caspase cascade and the cleavage of various caspase substrates such as caspase 3 and caspase 9 [9–11]. Morphologically, apoptosis is characterized by cellular and nuclear shrinkage as well as budding or blebbing which leads to the pinching off of blebs giving rise to “apoptotic bodies”, and chromatin condensation [10,11]. In addition, apoptosis is accompanied by internucleosomal DNA fragmentation giving rise to the classical “ladder” pattern on DNA electrophoresis [12,13]. In apoptosis, the functional integrity of the plasma membrane is long maintained.

Studies have shown that ATO induces apoptosis not only in leukemic and hematologic cells but also in solid tumors such as breast [14,15], neuroblastoma, [16]; murine lung [17–21], and bladder [22,23]. The apoptotic effects of ATO in these cell lines and solid tumors have been shown to be regulated through either the intrinsic or the extrinsic pathway. ATO has been found to be genotoxic in human cells such as pluripotent stem cells, keratinocytes, dendritic cells, and melanocytes [24, 25], leukemia cells [26], and hepatocellular carcinoma cells [27]. Arsenic compounds have been known to inhibit DNA repair, and induce chromosomal aberrations, sister chromatid exchanges and micronuclei formation in mammal cells. Several studies have been reported on the genotoxic potential of ATO and other arsenic compounds [26,27]. In vitro and in vivo studies that inorganic arsenic increases the frequency of micronuclei, chromosome aberrations, and sister chromatid exchanges in both animals and humans, however it does not induce point mutations [26–28].

Although studies on the effect of ATO on lung cancer cell lines are scarce, it is likely that this leukemia treatment drug may also have a bioactivity against lung cancer. Therefore, the study was designed to assess the potential for its use as a chemotherapeutic agent for the treatment of lung cancer; by investigating its oxidative, genotoxic and apoptotic mechanisms of action on A549 cells.

## Materials and Methods

### Cell line and chemicals

The human lung carcinoma cell line (A549), F-12 K medium, and trypan blue were purchased from American Type Culture Collection (ATCC) (Manassas, VA). The fetal bovine serum (FBS), penicillin/streptomycin/fungizone, phosphate buffered saline (PBS) and trypsin versene were purchased from Invitrogen (Grand Island, NY). ATO was purchased from Fisher Scientific (Houston, TX). P53 anti-mouse, bcl-2 anti-mouse, bcl-2 anti-rabbit, cytochrome c anti-mouse, hsp70 anti-mouse, caspase-3 anti-mouse and goat anti-mouse horseradish peroxidase (HRP) conjugated goat anti-rabbit (HRP) antibodies were purchased from EMD Biosciences (San Diego, CA). 4, 6-diamidine phenylindole (DAPI) stain was purchased from Invitrogen (Carlsbad, CA). Prolong gold antifade permount containing 1 µg/ml of DAPI was purchased from Molecular Probe (Eugene, OR). C-fos anti-rabbit antibody was purchased from Santa Cruz Biosciences (Santa Cruz, CA). ECL western blotting detection system reagents and film (CXposure) were purchased from Thermo Scientific/Pierce (Rockford, IL). The caspase 3-FITC and Annexin V-FITC assay kits were purchased from BD Pharmingen (San Diego, CA). The folin-phenol (DC) protein determination kit, cell lysis buffer, and non-fat milk were obtained from BioRad Laboratories (Hercules, CA). Polyvinylidene difluoride (PVDF) membrane, one-chamber glass slides, and comet assay kit from Millipore (Bedford, MA), (Lab-Tek Nunc, Naperville, IL), Trevigen Incorporation (Gaithersburg, MD), respectively.

### Cell culture

Human lung carcinoma (A549) cells were maintained in F12-K complete growth medium supplemented with 10% FBS and 1% penicillin (10,000 units/ml)/streptomycin (10,000 units/ml) (pen-strep) mixture as adherent cells. Cells were then grown in a humidified incubator under an atmosphere of 95% air and 5% CO2 at 37°C to sub-confluence (80–95%). The culture medium was replaced every 48 hr. After growing to 80–95% confluence, the medium was aspirated and the monolayer was washed three times with sterile phosphate buffered saline. The cell monolayer was treated with 1 ml 0.25% trypsin-0.5 mM EDTA per plate and incubated briefly at 37°C. The cells were then viewed microscopically to ensure a complete cell detachment. Cells were re-suspended in F12-K complete growth medium, stained with 0.4% trypan blue (1 to 2 min) and then counted with a hemacytometer. The cells were seeded at a density of 5 × 105 cells on 13 × 100 mm tissue cultured plates prior to treatment with arsenic trioxide.

### Nuclear staining with 4’, 6-diamidine phenylindole (DAPI)

Cell morphology was evaluated by Olympus 1X71 fluorescence microscopy following (DAPI) staining. The cells were cultured in one-chamber glass slides (Lab-Tek Nunc, Naperville, IL). After treatment with 0, 2, 4, and 6 µg/ml of arsenic trioxide for 48 hr, the slides were rinsed with PBS and fixed in PBS containing 3.7% paraformaldehyde for 30 min. After fixation, the slides were washed twice with PBS. Three drops of Prolong Gold anti-fade permount containing (1 µg/ml) of DAPI stain (Molecular Probe, Eugene, OR) were added to the slides and the slides were placed in the dark until later. The slides were then visualized using an Olympus Epifluorescence microscope equipped with a Spot Imaging camera (Diagnostic Instruments, Sterling, Height, MI) using the DAPI filter [29].

### Annexin V-FITC assay

To evaluate the effect of arsenic trioxide on early apoptosis, the Annexin V-FITC assay (BD Pharmingen) was performed according to a previous described protocol [30–33]. Briefly, A549 cells were seeded at a density of 3 × 10^5^ cells in F12-K complete medium on 13 × 100 mm tissue treated plates, and grown to 60–70% confluence. Cells were serum starved overnight in 1% FBS in F12-K medium supplemented with 1% penicillin/streptomycin. The serum medium was removed. The cells were reintroduced to F12-K complete medium, and treated with ATO at 0, 2, 4, and 6 µg/ml for 48 hr. The cells were washed twice with cold PBS and then resuspended in 1× binding buffer [10 mM Hepes/NaOH (pH 7.4), 140 mM NaCl, and 2.5 mM CaCl_2_]. The cells (100 µL) were transferred to a 5ml culture tube and 5 µL of Annexin V-FITC and 5 µL propidium iodide. The cells were vortexed and incubated for 15 min at room temperature (25°C) in the dark. Four hundred micro-liters of binding buffer (1×) was added to the tubes, analyzed and counted at 10,000 counts using a fluorescence-activated cell-sorting (FACS-Vantage) system (Becton-Dickinson, San Jose, CA).

### Caspase 3-FITC Assay

To assess the effect of arsenic trioxide on late apoptosis, caspase-3 assay was performed by flow cytometry according to previously described protocols [34–36] using a commercially available caspase-3 FITC assay kit (BD Pharmingen). A549 cells were seeded at a density of 3 × 10^5^ cells into F12-K complete medium on 13 × 100 mm tissue treated plate and grown to 60–70% confluence in 3 days. Sub-confluent cells were serum starved overnight. The cells were re-introduced to F12-K complete medium and treated with arsenic trioxide at 0, 2, 4, and 6 µg/ml, respectively for 48 hr. After exposure, cells were washed twice with cold PBS and resuspended in BD Cytofix/Cytoperm (neutral pH-buffed saline, saponin and 4% (w/v) paraformaldehyde) at a concentration of 1 × 10^6^ cells/ 0.5 ml. The cells were incubated for 20 min on ice. The cells were pelleted and the BD Cytofix/Cytoperm solution was aspirated and discarded. The cells were washed twice with BD Perm/Wash buffer at room temperature. The cells were resuspended in BD Perm/Wash buffer plus antibody and incubated for 30 min at room temperature. The pellets were resuspended in BD Perm/Wash buffer and analyzed and counted at 10,000 counts on the fluorescence-activated cell-sorting (FACS-Vantage) system (Becton-Dickinson, San Jose, CA).

### Preparation of cell lysates and western blot analysis

To assess the effects of ATO on protein expression, A549 cells were washed with PBS. The washed cells were lysed in a cell lysis buffer [20 mM Tris, 150 mM NaCl, 1 mM EDTA, 1 mM EGTA, 1% Triton X-100, 2.5 mM sodium pyrophosphate, 1 mM β-glycerophosphate, 1 mM Na_3_VO_4_, 1 µg/ml leupeptin and 1 mM phenylsulfonyl fluoride (PMSF)], and the lysates were centrifuged at 13,000 × g for 10 min. The supernatant was quantified and used for Western blot analysis. The protein concentrations were measured by folin-phenol (DC) protein reagent [37] using bovine serum albumin as a standard. Cell lysate containing 100 µg/20 µL of the protein was fractionated on 10% SDS-PAGE gel. The proteins were transferred onto a polyvinylidene difluoride (PVDF) membrane (Millipore, Bedford, MA) at a constant current of 400 mA overnight at 4°C. The membranes were blocked with 10% non-fat milk (Bio-Rad) in PBS containing 0.05% Tween-20 (PBST) for at least 1 hr at room temperature. The membranes were subsequently probed overnight at 4°C with anti-p53 ab-6 (1:500), anti-bcl-2 (1:250), anti-caspase 3 (1:1000), anti-Hsp 70 (1:1000), anti-cytochrome c (1:1000) primary antibody (EMD Biosciences, La Jolla, CA), and anti-cfos rabbit (1:1000) primary antibody (Cell Signaling Technology, Danver, MA) in primary antibody dilution buffer (1% nonfat milk in PBST). After the membranes were washed three times in PBST, they were incubated for 1 hr at room temperature with horseradish peroxidase (HRP) conjugated anti-mouse or anti-rabbit goat secondary antibodies (EMD Biosciences, La Jolla, CA) at a 1:10,000 dilution in PBST. The protein bands were detected with enhanced chemiluminescence (ECL-plus) western blotting detection system (GE Biosciences, Piscataway, NJ). Membranes were exposed to blue CXposure film (Thermo-Fisher Scientific, Houston, TX) and visualized by autoradiography using Kodak X-OMAT Processor (Mid-South Medical Imaging, Flowood, MS).

### Single cell gel electrophoresis (comet) assay

A549 cells were seeded on 13 × 100 mm tissue culture dishes at a density of 3 × 10^5^ cells per well in complete growth medium and grown to 70–75% confluence in a humidified incubator under an atmosphere of 95% air and 5% CO_2_ at 37°C. Sub-confluent cells were incubated in 1% fetal bovine serum supplemented with 1% antibiotic for 24 hr prior to treatment. Afterward, the cells were reintroduced into complete growth medium supplemented with 0, 2, 4, and 6 µg/ml of arsenic trioxide for 48 hr. Cells incubated in complete growth medium served as a control. After 48 hr, the comet assay was performed following the manufacture protocol [38]. Following ATO treatment, the medium was removed and the cells were washed three times with PBS, trypsinized with 1ml of 0.25% trypsin-EDTA, harvested, and counted. The cells were spun down at 3000 rpm for 5 min. The pellet was resuspended in PBS at a cell density of 1×105. The cells were combined with molten LMAgarose (42°C) at a ratio of 1:10 (v/v), and 75 µl was immediately pipetted onto Cometslide™. The comet slides were placed flat in a 4°C refrigerator for 30 min and then immersed in pre-chilled lysis solution on ice for 1 hr. The lysis buffer was removed, and the slides were immersed in freshly prepared alkaline solution, pH > 13 for 1 hr. Slides were washed twice for 5 min with 1× TBE (Tris boric acid EDTA) and electrophoresed at 1 volt/cm (22v) for 10 min. Slides were placed in 70% ethanol for 5 min. The excess ethanol was removed. Slides were air dried overnight, stained with SYBRgreen, and allowed to set for 12 hr. The comet slides were viewed and analyzed using the Olympus Epifluorescence Microscope with LAI’s Automated Comet Analysis Scoring System software (Loates Associates, Inc. Westminster, MD). A total of 150 comets were scored per arsenic trioxide dose. Seventy-five comets were randomly selected from three replicated slides. The experiment was repeated three times.

### Statistical analysis

Experiments were carried out in triplicates, and the data were presented as means ± SDs. To test for differences among and between experimental groups, one–way analysis of variance (ANOVA) and Student’s t-test were performed respectively, using SAS software available in the Biostatistics Core Laboratory available at the RCMI Center for Environmental Health at Jackson State University for testing differences. Data were considered statistically significant for p-values less than 0.05.

## Results

Effect of arsenic trioxide on morphological and apoptotic-related changes in A549 cells.

**Figure 1:**
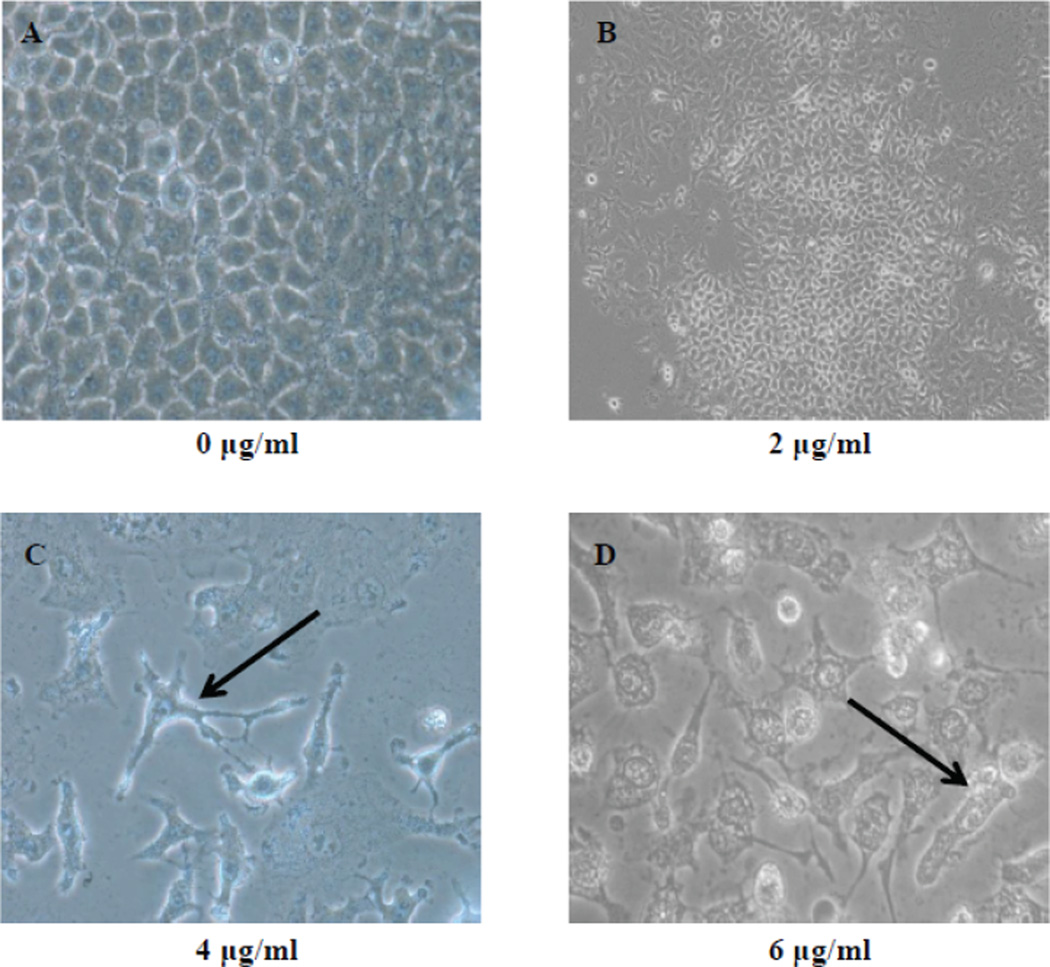
Morphological changes of A549 lung cancer cells after 48 hr ATO exposure. A-Untreated control; B – 2 µg/ml ATO; C – 4 µg/ml ATO; and D - 6 µg/ml ATO. A549 cells were observed using an Olympus Inverted Phase Contrast Microscope with camera (C-Squared), at (20×) magnification for Panel A and Panel B, and (40×) magnification for Panel C and Panel D. Cells in Panels A and B maintained normal morphological features. The black arrow shows an example of cell shrinkage in Panel C, and an evidence of chromatin condensation and membrane blebbing in Panel D; bo`th of which are indicative of apoptosis.

There are classical changes on cells that undergo apoptosis. Cell shrinkage, membrane blebbing and chromatin condensation were identified as the morphological hallmarks for apoptosis. We examined apoptotic features of A549 cells treated with ATO and untreated A549 cells. The phase contrast micrograph of untreated A549 cells revealed healthy cells also the micrograph revealed round distinctive nuclei with intact cytoplasm (Figures 1A–B). The phase contrast observation revealed that A549 cells treated with ATO showed evidence of cell shrinkage (Figure 1C), membrane blebbing and condensed chromatin (Figure 1D).

**Figure 2:**
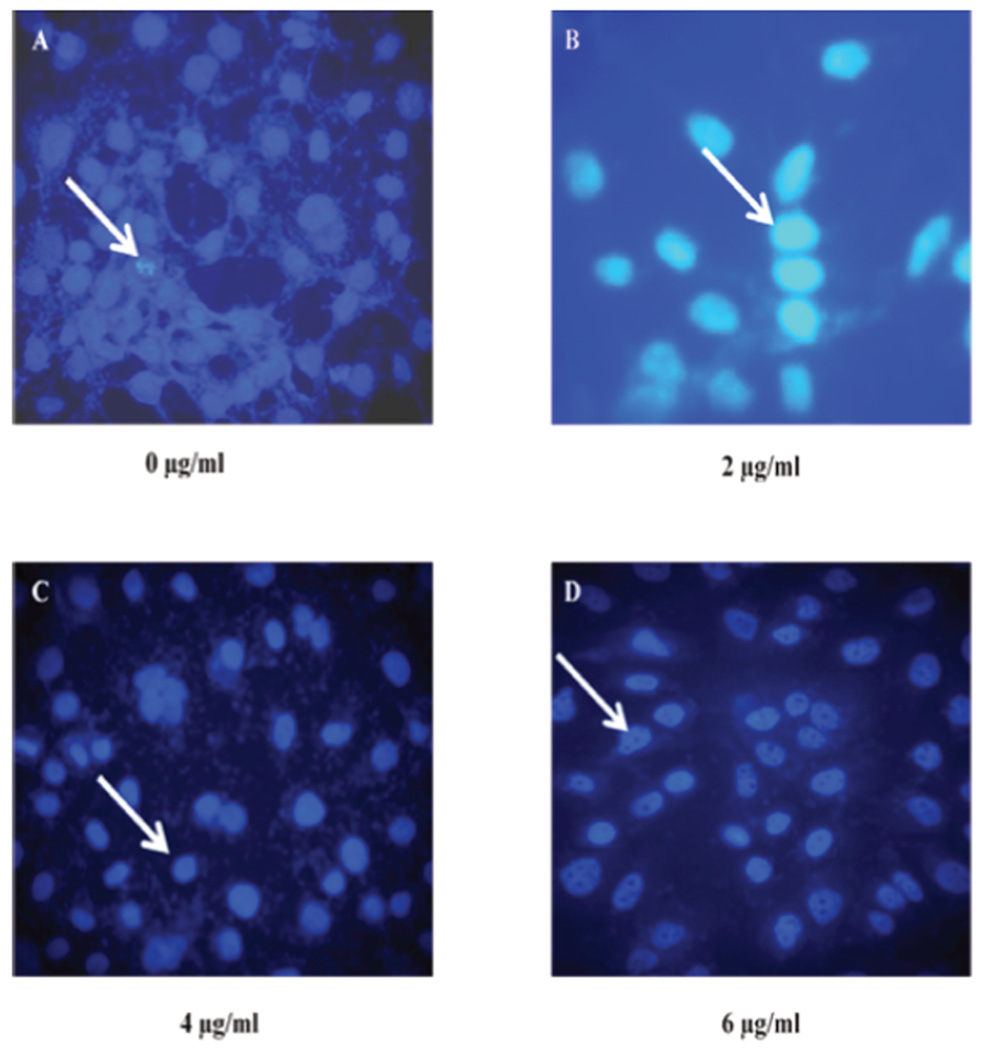
Effect of ATO on morphological and apoptotic-related changes in A549 lung cancer cells. The cells were stained with DAPI and visualized on an Olympus Epifluorescence microscope 20×. A549 cells were treated for 48 hr with ATO at 0 (control), 2, 4 and 6 µg/ml respectively. Panel A (0 µg/ml), the arrow points to a nucleus fluorescing bright blue; Panel B (2 µg/ml), the black arrow indicates viable cells and the white arrow represents a non-viable cell; Panel C (4 µg/ml), the arrows indicate apoptotic nuclei with condensed chromatin; and Panel D (6 µg/ml), the arrow is pointing to apoptotic bodies within the nucleus.

The nuclear morphology changes were assessed by DAPI staining. DAPI permeates the plasma membrane and yields blue chromatin Viable cells displayed normal nuclear size and blue fluorescence as shown in Figure 2A. ATO-treated cells showed evidence of apoptosis as characterized by the cell nuclei undergoing fragmentation forming apoptotic bodies and the chromatin being condensed (Figures 2B–D).

### Effect of arsenic trioxide on early apoptosis

**Figure 3:**
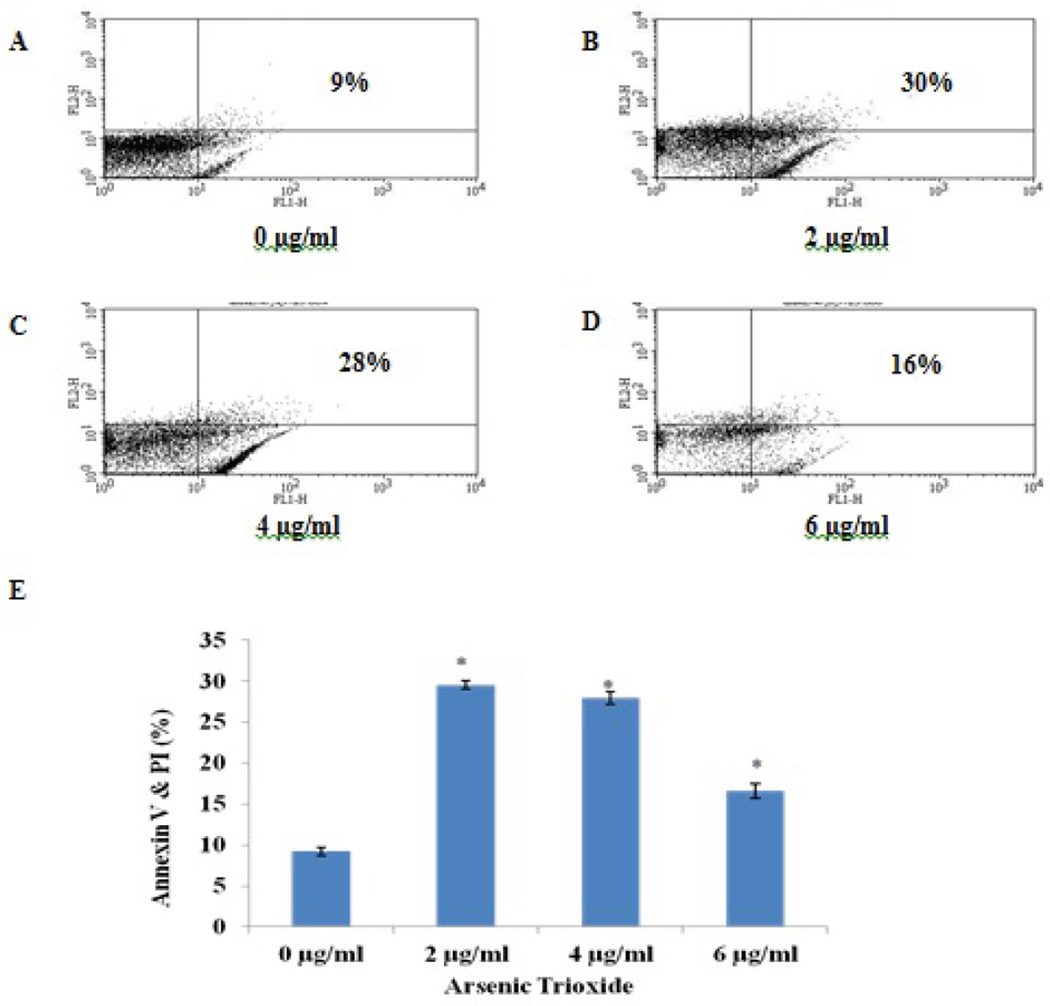
Effect of arsenic trioxide on early apoptosis. Arsenic trioxide induced apoptosis in A549 cells. Propidium iodide (PI) is represented by FL1-H on the x-axis, and annexin V-FITC is represented by FL2-H on the y-axis. Viable cells are found in the lower left quadrant (annexin V and PI negative), apoptotic cells are found in the upper left quadrant (annexin V positive and PI negative), dead cells are found in the upper right quadrant (annexin V and PI positive), and damaged cells are found in the lower right quadrant (annexin V negative and PI positive). Annexin V conjugated with propidium iodide (PI) revealed dead cells as represented in upper right quadrants (A–D). Data from the Annexin V FITC flow cytometry analysis of ATO 48 hr exposure revealed that the dead cells were significantly different (*) compared to the control (E) using the one-way ANOVA analysis (*p* < 0.05).

To assess the effect of arsenic trioxide on early apoptosis in A549 cells treated with ATO, Annexin V-FITC assay was performed. Annexin V binds to the membrane phospholipid phosphatidylserine that is located within the plasma membrane of apoptotic cells. Annexin V positive and propidium iodide negative cells were considered to be apoptotic. The histograms in Figure 3A are representations of cell populations. The viable cell populations are represented in the lower left quadrant of the histogram as Annexin V negative and PI negative and the lower right quadrant of the histogram represents apoptotic cells as Annexin V positive and PI negative. The percentages of viable cells were 89%, 56%, 66%, and 40% for 0, 2, 4, and 6 µg/ml, respectively (Figures 3A–D). The percentages for apoptotic cells were 9.0 ± 0.50, 30.00 ± 0.50, 28.00 ± 0.70 and 17.00 ± 0.90 for 0, 2, 4 and 6 µg/ml, respectively.

### Effect of arsenic trioxide on late apoptosis

**Figure 4:**
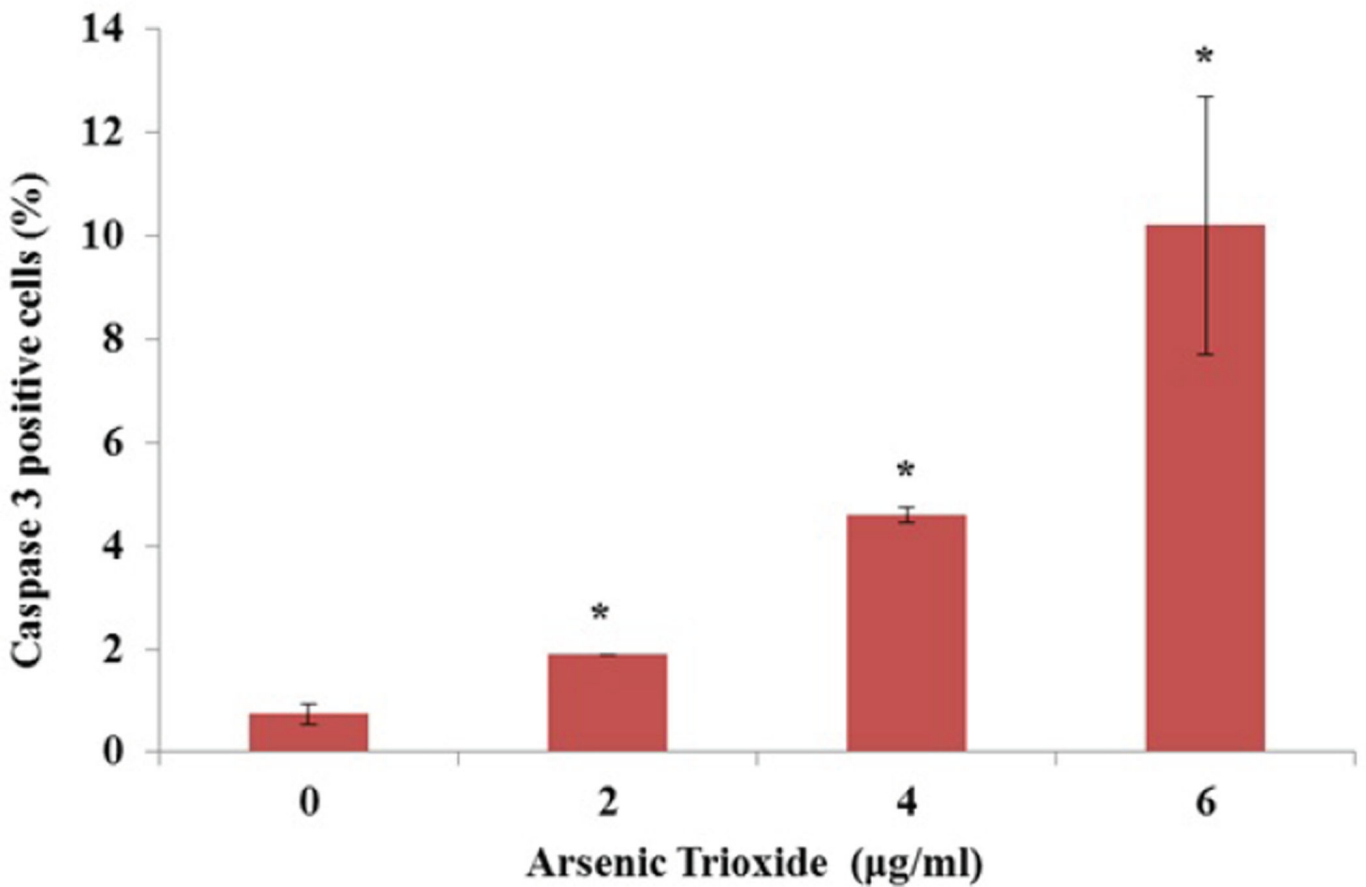
Effect of arsenic trioxide on caspase 3 activity in A549 cells after 48 hr treatment. The data is represented as mean ± SEM of three experiments performed in triplicates. The differences in mean percentages were considered statistically significant with a p value < 0.05. The significance of the value is indicated by asterisks (*).

To examine whether caspase-3 was activated during arsenic trioxide induced apoptosis, a caspase-3 FITC assay was performed. As shown in Figure 4, the flow cytometric data revealed that the percentages of caspase-3 positive cells were 0.74 ± 0.19%, 1.90 ± 0.00%, 4.60 ± 0.14% and 10.20 ± 2.50% for 0, 2, 4, and 6 µg/ml ATO, respectively. Statistically significant differences (p < 0.05) in caspase-3 activity were observed at 4 and 6 µg/ml of ATO when compared to the control.

### Effect of arsenic trioxide on the expression of apoptotic and stress proteins

**Figure 5:**
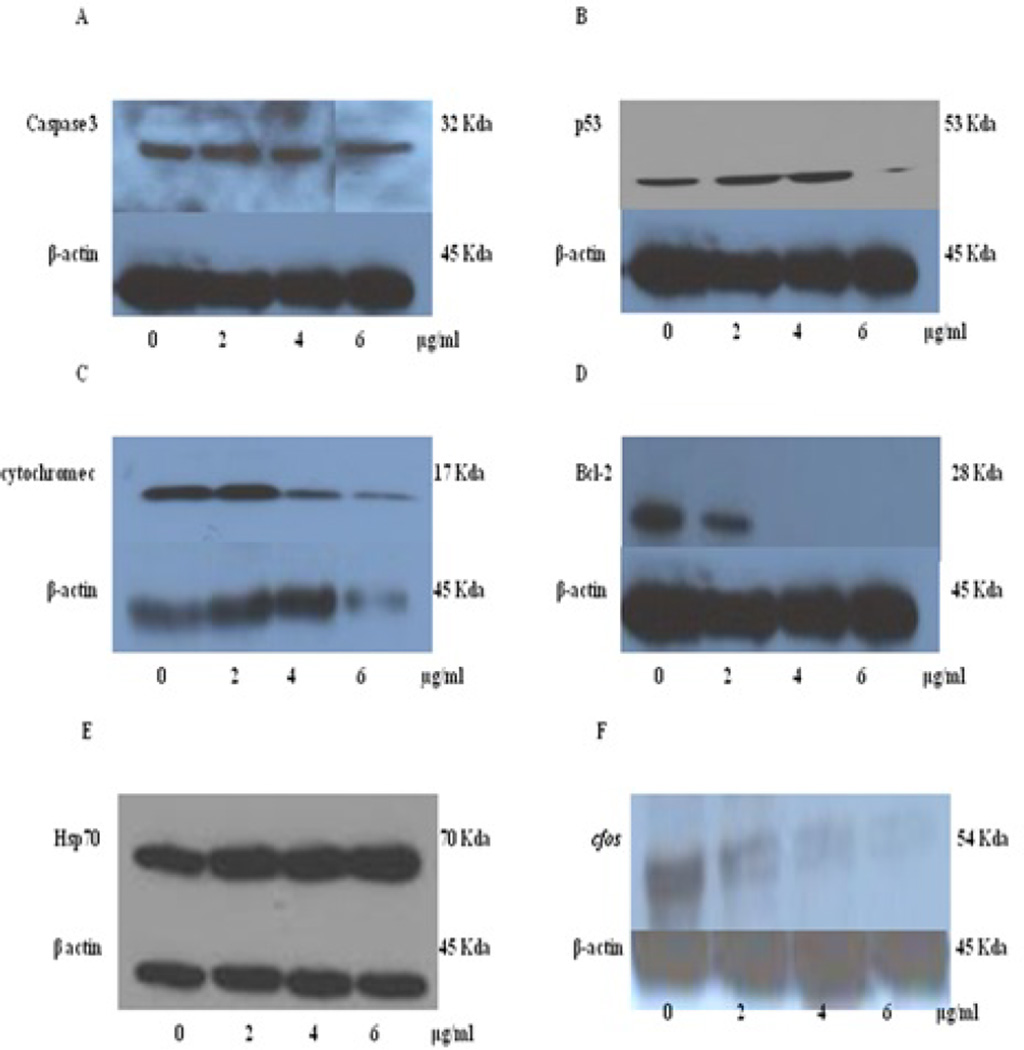
Western blot analysis of expression of apoptotic proteins (A – D) and stress proteins (E – F) ATO-treated A549 cells after 48 hr of exposure. The figures represent: A– caspase 3 expressions; B – p53 expression; C – cytochrome c expression; D – Bcl-2 expression; E – Hsp 70 expression; and F – cfos expression.

To validate that arsenic trioxide induced the expression of caspase-3, p53, bcl-2, and cytochrome c proteins by Western blot analysis. Study results indicated that caspase-3 was activated in a dose dependent manner to ATO (Figure 5A). The p53 protein is a determinant in controlling the cell cycle and apoptosis. The p53 protein expression in Figure 5B increased in a dose dependent apoptosis, we evaluated manner between 0 and 4 µg/ml. There was a slight down-regulation of p53 expression at 6 µg/ml of ATO probably due to the high percentage of cell death at higher level of ATO treatment. Western blot analysis revealed that cytochrome c expression substantially increased at 2 µg/ml and down-regulated at 4 and 6 µg/ml of ATO (Figure 5C). Bcl-2 expression was significantly decreased in a dose-dependent manner with response ATO treatment (Figure 5D).

To assess whether ATO induces oxidative stress, we tested the expression of Hsp70 and cfos stress proteins. The western blot analysis revealed a dose-dependent up-regulation of Hsp70 with increasing ATO doses from 0 to 6 µg/ml. This was indicative of cells undergoing oxidative stress (Figure 5E). On the other hand, a dose dependent decrease was observed with regard to c-fos expression (Figure 5F).

### Genotoxic effects of arsenic trioxide

**Figure 6:**
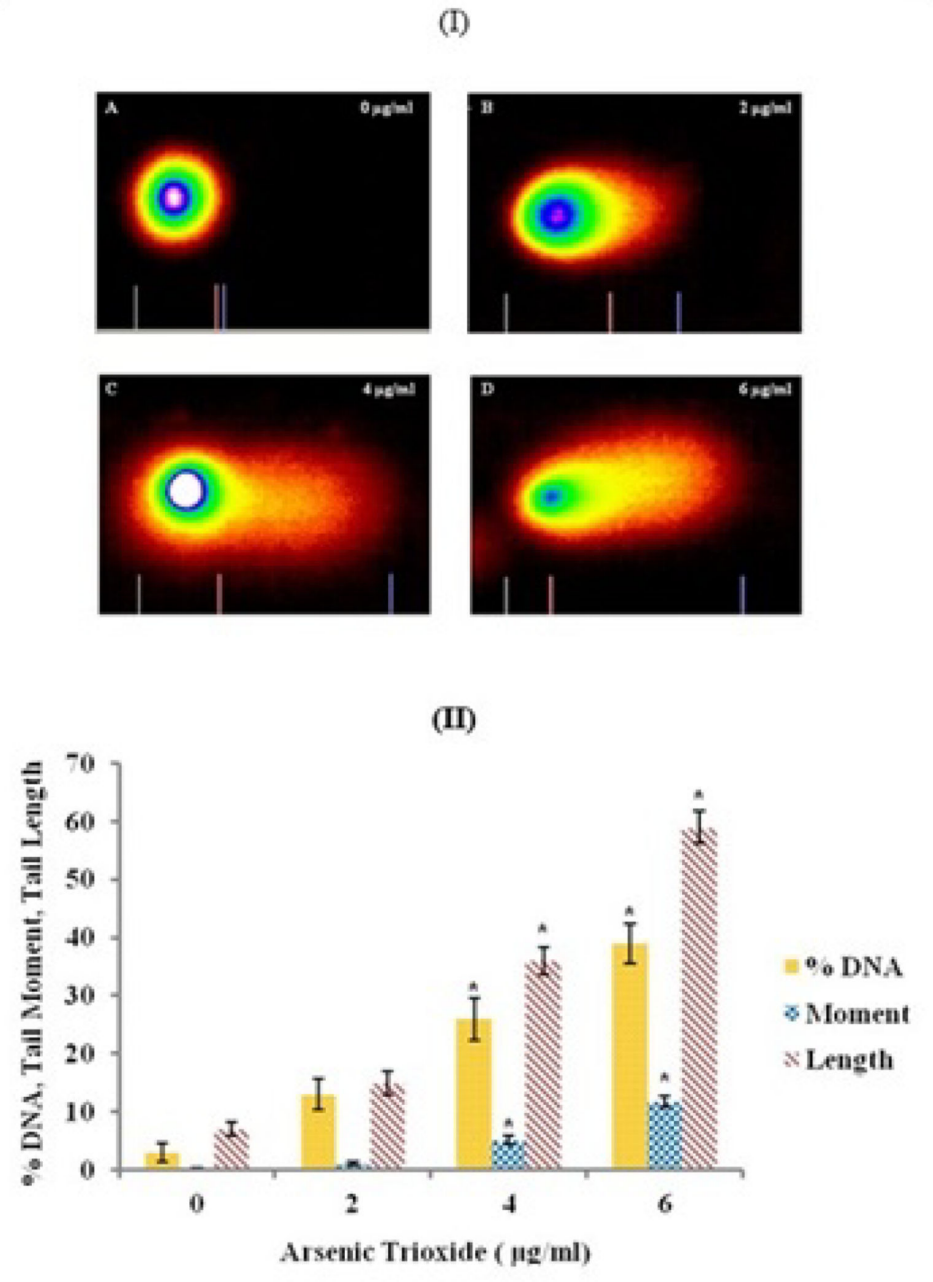
(I) Representation of Comet assay images of A549 cells treated with ATO at 0 µg/ml (A); 2 µg/ml (B), 4 µg/ml (C), and 6 µg/ml (D) after 48 hr of exposure. (II) DNA damage in A549 cells treated with arsenic trioxide. The figure shows the percentages of DNA damage, olive tail moment and Comet tail length.

To assess the effect of ATO on genotoxicity in A549 cells, single-cell gel electrophoresis (Comet) assay was used to evaluate DNA damage. Comet images in Figures 6A–D displayed the cell DNA migration patterns in A549 cells treated with 0, 2, 4, and 6 µg/ml of arsenic trioxide, respectively. The comet tail lengths, percentages DNA damage and olive tail moment were calculated. As shown in Figure 6I (A), the nuclear DNA of untreated cells was perfectly round and retained a highly organized association with matrix proteins in the nucleus. The nuclear DNA of ATO-treated cell was severely fragmented as the dosage increases. Also, the cellular organization was disrupted as depicted Figure 6I (B–D).

The Comet assay data generated from three separate experiments were analyzed and the mean values of DNA damage, Comet tail length and Comet tail moment were graphically illustrated (Figure 6II). The results show a significant dose-response relationship with regard to ATO-induced genotoxicity. The percentages of DNA damage were 3.0 ± 1.5%, 13 ± 2.7%, 26 ± 3.6%, and 39 ± 3.4% for 0, 2, 4 and 6 µg/ml of ATO, respectively. The data for Comet tail moment were 0.19 ± 0.07, 1.06 ± 0.35, 5.1 ± 0.66 and 11.7 ± 0.95 for 0, 2, 4 and 6 µg/ml of ATO, respectively. The data for Comet tail lengths were 7 ± 1.2 µm, 15 ± 2.0 µm, 36 ± 2.3 µm and 57 ± 2.7 µm for 0, 2, 4 and 6 µg/ml of ATO, respectively.

## Discussion

Apoptosis assessment is a gene directed tool for understanding developmental biology and tissue homeostasis [39]. In previous study, we demonstrated that ATO has the potential to induce apoptosis in breast cancer and lung cancer cells [40]. In the present study, we observed characteristic apoptosis-related morphological changes in lung cancer cells (A549) exposed to ATO; as shown on the phase contrast micrographs (Figure 1C and 1D) and on the fluorescence micrographs (Figures 2A–D). Furthermore, we also observed a significant increase in the level of externalization of the plasma membrane molecule phosphatidylserine (Figure 3). Li et al. [41] reported other morphological changes in the colon, breast, and pancreatic cancer cell lines after exposure to ATO. These changes included reduced cytoplasmic volume, membrane blebbing, formation of apoptotic bodies and nuclear condensation consistent with apoptosis. To gain an insight into the molecular mechanisms involved in apoptosis caused by ATO in A549 cells, we further evaluated the expression levels of apoptotic–related proteins including caspase 3, p53 and cyctochrome c.

Caspases are a family of proteases which play a pivotal role in the execution of apoptosis [10]. Caspase-3 was used as a biomarker for apoptosis in this study. It was observed that caspase-3 activity increased in ATO-treated cells a dose-dependent manner (Figure 4A). Previous studies have shown that caspase-3 plays an essential role as an executor of apoptosis [11,42]. Han et al. [17] observed a significant increase of caspase-3 activity in juxtaglomerular cells (As4.1 JG) treated with 7 µM arsenic trioxide for 48 hr compared to control. To confirm the role of caspase 3 in ATO-induced apoptosis, these investigators also tested the response of ATO-treated As4.1 JG cells in the presence of caspase-3 inhibitor [17,42]. By Western blot analysis, Shim et al. [43] reported that arsenic trioxide (10 µM) induced apoptosis through caspase-3 activation in chronic myelogenous leukemia (K562) cells.

It has been pointed out that caspase-3 is activated by caspase-9 via activation of BID by caspase-8, loss of mitochondrial membrane potential (Δψm) and cytochrome c release in the cytosol. This is called the mitochondrial apoptotic pathway [44]. In the present study, we found an up regulation of cytochrome c at 2 µg/ml and a down-regulation at 4 and 6 µg/ml ATO (Figure 5C). We also found that there was a strong dose-dependent down regulation of bcl-2 protein expression in A549 exposed to ATO (Figure 5D). This finding is consistent with other studies reporting the involvement of bcl-2 cleavage in the acceleration of chemical-induced cell apoptosis [45]. Contrary to our results Nakagawa et al. [45] observed that colon cancer cells (SWAs) treated with 2 µM of arsenic trioxide for 72 hr did not undergo apoptosis. However, they reported in another study that other colon cancer cells (SW480) underwent apoptosis following exposure to ATO at similar treatment dose and time period [46]. They also pointed out that the over-expression of bcl-2 protein could not completely prevent apoptosis induced by ATO. These investigators concluded that arsenic trioxide-induced apoptosis was not mediated via the mitochondria [46,47]. From the present study, we believe the arsenic trioxide–induced apoptosis was mediated through the mitochondria pathway which activated caspase 3.

The p53 protein is a tumor suppressor gene or protein that controls multiple functions in biologic systems. It has been implicated in the mechanism by which arsenic induces cell-cycle arrest, and DNA damage [48]. The p53 gene has been mapped to chromosome 17 [49] and play a vital role in apoptosis and check point control both at G_1_/S and G_2_/M phases in response to DNA damage [50–52]. The data from our western blot analysis revealed that the p53 protein expression significantly increased from 0–4 µg/ml as shown in Figure 5B. However, our results revealed a slight decrease of p53 expression at 6 µg/ml of ATO (Figure 5B). Hans et al. [17] observed that p53 expression dramatically increased in the AS4.1 renal cells treated with 1 µM of arsenic trioxide [17]. Lui et al. [53] reported that multiple myeloma cells with normal p53 were resistant to arsenic trioxide–induced apoptosis and were arrested in G_1_ phase.

Stress or heat shock proteins (HSPs) are expressed in response to a wide variety of physiological and environmental insults such as heat, reactive oxygen species or anticancer drugs [54]. The elevated levels of Hsp70 proteins have been linked with inhibition of apoptosis. Cells resistant to chemotherapeutic agents have been associated with elevated levels of Hsp70 proteins. To further examine that ATO induced oxidative stress, the expression of Hsp70 and cfos stress proteins was examined. The western blot analysis revealed a dose response relationship with regard to ATO treatment. There was a strong up-regulation of Hsp70 in ATO-treated cells compared to the control (Figure 5C). This is indicative of the cells undergoing oxidative stress or inflammatory reaction [55,56].

The c-fos protein is the product of c-fos mRNA, a member of a family of immediate early gene (IEG) transcription factors (other members include Jun and Egr-1) also identified as proto-oncogenes. It has been discovered in mutated and oncogenic forms in mouse osteosarcomatogenic retroviruses [57]. These transcription factors are involved in the control of proliferation, differentiation and apoptosis, as well as in the control of responses to stress, and play an important role in organogenesis [58]. Several researchers have reported that arsenite activated the transcription factor Ap-1, as a consequence of increasing the activity of its mitogenic component (cfos and c-jun) [59,60]. The cfos western blot results revealed a downregulation at 2–4 µg/ml of arsenic trioxide at 48 hr treatment in (Figure 5F). Our data did not agree with result from Chen and others [42], who reported that low levels of arsenites induced proliferation by over expression of cfos protein [42]. We believed the downregulation of cfos at 2–4 µg/ml of arsenic trioxide is a stress response induced by arsenic trioxide in the lung (A549) cells. We did not observed cell proliferation at these levels of exposure.

The single cell gel electrophoresis (comet assay) is an assessment tool used to measure single-strand, double-strand DNA breaks and DNA cleavage in mammalian cells [38]. The length of a comet tail (tail migration), olive tail moment and percent DNA are evidence of DNA damage using the alkaline comet assay. In this study, we used this assay to study DNA damage after 48 hr of A549 cells to arsenic trioxide. The results revealed a dose-dependent increase in percentages of DNA damage, Comet tail length and olive tail moment Figures 6I (A–C). Graham et al. [24] reported an increase in Comet tail length and Comet tail moment after 24 hr exposure of human induced pluripotent stem cells to ATO. Recent study in our laboratory also reported similar findings with colon (HT-29) cells treated with arsenic trioxide for 24 hr [61]. However, the data revealed DNA damage at a higher level of ATO exposure in colon cancer cells compared to lung cancer cells. Hence, lung cancer cells appear to be more sensitive than colon cancer cells to DNA damage caused by arsenic trioxide.

Several studies have shown that ATO is genotoxic, causing DNA damage to human leukemia (HL-60) cancer cells in a dose-dependent manner [26,44,62]. Graham et al. (2003) reported that arsenic was highly genotoxic to human keratinocytes, melanocytes, and dendritic cells. Studies have also shown that arsenic-induces DNA damage in human hepatocytes and urothelial cells measured by the comet assay [63] as well as in lymphocytes [64]. Using the alkaline comet assay, Guillamet et al. [65] also reported DNA damage in human lymphoblastoid (TK6) cell exposed to arsenic-containing compounds.

## Conclusions

In conclusion, the results of this research demonstrate that arsenic trioxide causes significant toxicity to lung carcinoma (A549) cells, and its toxic effects seem to be mediated through oxidative, apoptotic and genotoxic mechanisms. The findings in this study also suggest that ATO has the potential to be used as a chemotherapeutic agent in the treatment of non-small cell lung cancer. However, further in vivo studies using animal models of lung tumoriogenesis are needed to confirm the therapeutic spectrum of arsenic trioxide.
